# Trends in the prevalence of concurrent anaemia and stunting among infants and young children in Rwanda: a cross-sectional study from 2010 to 2020

**DOI:** 10.1080/16549716.2025.2466281

**Published:** 2025-02-19

**Authors:** Mathieu Nemerimana, Mojeed Akorede Gbadamosi

**Affiliations:** a School of Public Health, Mount Kigali University, Kigali, Rwanda; b School of Medicine and Pharmacy, College of Medicine and Health Sciences, University of Rwanda, Kigali, Rwanda

**Keywords:** Infant nutrition, nutritional deficiencies, comorbid anaemia and stunting, undernutrition, Demographic and Health Surveys, Rwanda

## Abstract

**Background:**

Concurrent anaemia and stunting (CAS) pose severe public health risks in low- and middle-income countries, affecting child health and development.

**Objectives:**

To determine trends in CAS prevalence and associated factors among infants and young children aged 6–23 months in Rwanda.

**Methods:**

Analyses of nationally representative samples from the Rwanda Demographic and Health Surveys (RDHS) of 2010, 2014/15, and 2019/20 were conducted. Participants’ characteristics, trends, and prevalence of CAS were analysed using frequencies and percentages. Multivariable binary logistic regression analyses were used to assess factors associated with CAS.

**Results:**

The prevalence of CAS among children aged 6–23 months in Rwanda declined from 21.3% in 2010 to 16.9% in 2019/20 (*p* = 0.005). Significant factors associated with CAS included child’s age above 12 months, male sex, small/very small birth size, breastfeeding initiation 1 day post-birth, inadequate minimum acceptable diet, history of cough 2 weeks prior to the survey, multiple births, being from a mother with no or only primary education, mother aged below 20 years, mother with no iron supplementation during pregnancy, maternal anaemia, resident of an eastern province, resident of high altitude areas (>2000 m), low household wealth and unimproved toilet facilities.

**Conclusions:**

This study reveals persistent CAS among infants and young children in Rwanda. CAS was associated with various child, maternal, and household-related factors. Despite a notable decline in CAS prevalence in Rwanda over the past decade, CAS remains a significant public health issue, requiring targeted interventions.

## PAPER CONTEXT

- **Main findings**: There was a significant decline in the prevalence of concurrent anaemia and stunting among Rwandan children aged 6–23 months from 2010 to 2019/2020. However, the rates remain high and warrant continued attention.
- **Added knowledge**: Undernutrition remains a major public health concern in many developing countries, including Rwanda. This study provides comprehensive evidence on the magnitude of and factors associated with the concurrence of anaemia and stunting in Rwandan children.
- **Global health impact for policy action**: As in many developing countries, greater efforts are needed to ensure that existing strategies and interventions to prevent malnutrition address the coexistence of multiple forms of nutritional deficiencies in infants and young children.

## Background

Ending all forms of malnutrition in children by 2030 is a critical objective of the second United Nations Sustainable Development Goal [1]. The World Health Organization has set a target to reduce childhood stunting by 40% by 2025 [2]. Despite these ambitious goals, anaemia and stunting remain significant public health challenges worldwide [3–5]. Globally, approximately 40% of children under five are affected by anaemia [3,6], and over 22% are stunted [5]. The situation is particularly difficult in Sub-Saharan Africa, where 64% of children suffer from anaemia [7], and about 30% of the world’s stunted children reside in the region [5]. In Rwanda, the rates of anaemia and stunting among children remain alarmingly high; 37% of children under five are anaemic, and one in three are stunted [8]. Among malnourished children in Rwanda, the anaemia rate is even higher, with approximately 69% being anaemic [9]. Anaemia and stunting each independently increase the risk of mortality and morbidity in children. They are also linked with long-term adverse effects including impaired cognitive and physical development, growth failure, increased vulnerability to chronic diseases, and poor academic performance in later life, ultimately depressing economic productivity [10–15]. The coexistence of anaemia and stunting in a child is associated with detrimental effects on health and development. These adverse consequences are exacerbated when the child is simultaneously affected by both conditions during the first 24 months of life [10].

Most forms of undernutrition have overlapping risk factors, particularly for basic and underlying determinants [16,17], including inadequate nutrient intake, inappropriate feeding practices, childhood illnesses, inadequate hygiene and sanitation, low socio-economic status, and food insecurity [17]. Similarly, the causal factors for anaemia and stunting are multi-dimensional and interlinked, making it likely that a child affected by either condition will be affected by both [18].

Globally, various studies have shown that the prevalence of concurrent anaemia and stunting (CAS) in children is variable from countries, with high rates in developing countries [10,18–20]. For instance, a study across 22 Sub-Saharan African countries found that 25% of children are affected by CAS [19]. In Ethiopia, the prevalence of CAS among young children was 24% [20], while in Peru and India, the rates were 22% and 30%, respectively [10]. Main factors associated with CAS across different countries included poor child feeding practices, small birth size, history of infections, low caregivers’ education and age, poverty, and poor household conditions [19,20].

In Rwanda, there is limited evidence on the prevalence of CAS in children. While global and regional studies provide insights into CAS, specific factors driving this condition in Rwanda are largely unknown. Thus, context-specific evidence is essentially needed to inform interventions. Several studies conducted in Rwanda have focused on assessing separate factors associated with anaemia and stunting [9,21–25] among children, yet the concurrence of anaemia and stunting has been largely overlooked in Rwanda. This gap underscores the need for studies to provide evidence of the magnitude of CAS and its predictors in infants and young children. In settings such as Rwanda, where childhood anaemia remains prevalent, despite a slow reduction in stunting rates [8], there is an increased likelihood of children presenting with syndromic conditions with multiple nutritional deficits. This study used secondary data collected from the Rwanda Demographic and Health Surveys (RDHS). The aim was to determine trends in prevalence and factors associated with CAS among infants and young children aged 6–23 months and to assess prevalence trends from 2010 to 2020.

## Methods

### Study setting

Rwanda is a landlocked country in East Africa that has 30 administrative districts located in four provinces and in the city of Kigali. According to a recent national census on population and housing, the population of Rwanda is 13,246,394 in 2024 [26].

### Study design and sample

This study used an open-access dataset of RDHS of 2010, 2014/2015, and 2019/2020 [8,27,28]. The RDHS is a national cross-sectional survey conducted every 5 years by the National Institute of Statistics of Rwanda in partnership with the Rwanda Ministry of Health [8,27,28]. All three national surveys used two-stage sampling designs. Stage one of the sampling approach consisted of sampling the villages (clusters or enumeration areas). For effective national representation, 492 clusters were chosen for RDHS 2010, 492 clusters were chosen for RDHS 2014/2015, and 500 clusters were chosen for RDHS 2019/2020. The second stage involved systematic sampling to select the households. In this second stage, 12,570 households, 12,793 households, and 13,000 households were selected from chosen clusters to have national representation for RDHS 2010, 2014/2015, and 2019/2020, respectively. Among these households, 2372 children, 2409 children, and 2363 children aged 6–23 months were selected for RDHS 2010, 2014/2015, and 2019/2020, respectively [8,27,28]. More details of the protocols describing for data collection methods can be found elsewhere [8,27,28]. All children aged 6–23 months with available data on anaemia and stunting status, were included in the study population. This age range was chosen for the following reasons. First, children aged under 2 years are highly vulnerable to developing anaemia [11]; second, most stunting occurs during this first early period of life, and third, data required including feeding practices and micronutrients intake are only available for this age category in the RDHS.

### Description of variables

The main outcome, CAS, was defined as when a child has both anaemia and stunting at the same time. This was a binary or dummy variable, i.e. having CAS or not. Anaemia was defined as having blood haemoglobin levels less than 11 g/dL (8,27,28) and stunting is defined by length/height-for-age Z-scores less than −2 [8,27–29].

The independent variables, described below, were selected based on previous similar studies conducted on CAS [10,18,20,30]. Child age was categorized into 6–11 months and 12–23 months. Child sex was female or male. Birth size, classified as very small, small, and average or larger, was derived from the mother’s subjective reporting of birth size [8,27–29]. Breastfeeding initiation after birth was also based on the mother’s subjective report. The variable was categorized as within the first hour, one to 14 h, and one to 30 days after birth. The variable ‘currently breastfeeding’ refers to the child’s breastfeeding status at the time of the survey. This was a binary variable ‘yes’ or ‘no’.

Minimum dietary diversity (MDD) was defined as when a 6–23-month-old child had reportedly taken a minimum of five or more of eight recommended food categories in the 24 h prior to the survey interview [31]. This was a binary variable, ‘adequate MDD’ versus ‘inadequate MDD’. Minimum meal frequency (MMF) was defined as when a 6–23-month-old child received complementary foods at least the minimum number of times during the previous day. Minimum recommended number is 2 times for breastfed infants aged 6–8 months, 3 times for breastfed infants aged 9–23 months and ≥4 times for non-breastfed infants aged 6–23 months [31]. The variable MMF was binary and coded as adequate MMF versus inadequate MMF. Minimum acceptable diet (MAD) was defined as when a child aged 6–23 months has eaten a varied diet with at least five groups of food at minimum recommended meal frequency in the previous 24 h [31]. This variable was also coded as adequate versus inadequate. If the mother reported that the child had been given vitamin A rich fruits during last 24 h of the survey, this was coded as ‘yes’ or ‘no’.

The variable, which referred to whether the child had received drugs for intestinal parasites in the 6 months prior to the survey interview, was binary and coded ‘yes’ or ‘no’. Variables referring to diarrhoea, fever, or cough in the 2 weeks prior to the survey interview were also binary. Type of birth was categorized as singleton or multiple births. Mother’s age was categorized into three groups (<20, 20–34, and 35–49 years). Mother’s educational status was categorized as no education, primary and secondary, and higher. Maternal body mass index (BMI) was categorized as underweight (BMI <18.5), normal (BMI ≥18.5 & ≤24.9), overweight (BMI >24.9 & ≤29.9), and obese (BMI >29.9). Maternal anaemia status was binary, as were variables that identified whether the mother reported having had iron supplementation and drugs for intestinal parasites during the pregnancy in question. Place of residence was categorized as ‘urban’ or ‘rural’. Cluster altitude was categorised as <1500 m, 1500–2000 m, and >2000 m. The household wealth index is derived from a principal component analysis of data on household ownership assets, and was categorized in quintiles – poorest, poorer, middle, richer and richest [31]. Source of household drinking water and the type of household toilet facilities were classified as ‘improved’ and ‘not improved’. All the variables were generated and coded according to DHS guidelines [31].

### Statistical analysis

Data cleaning and analysis were conducted using Stata V.18.1. Following the DHS standards, which explain how to identify codes and recode variables [31] and based on the inclusion criteria for this study, each dataset for the RDHS 2010, 2014/2015, and 2019/2020 was independently cleaned

Stata svy commands were used to adjust for survey design and standard errors related to cluster sampling and sampling weight. Participants’ characteristics were described using descriptive analysis and presented using frequencies and percentages. The prevalence of CAS for each RDHS was calculated based on weighted data using a subpopulation of children aged 6–23 months. Descriptive analysis was conducted to identify trends in prevalence rates of CAS. The Cochran-Armitage test for trend was used to assess the significance of changes in CAS prevalence over time. The Marascuillo procedure was applied to determine whether the differences between pairs of prevalence rates were statistically significant [32]. Using this procedure [32], the three pairs of the prevalence rates of CAS in the three RDHS were compared to obtain the value of the absolute difference and determine the critical range. The difference between the two prevalence rates was considered statistically significant if the value was higher than the critical range [32]. Multivariable binary logistic regression analysis was conducted to assess significant factors associated with CAS. Only variables from the bivariate analysis with a p-value below 0.05 were included in the multivariable logistic regression analysis. Statistical significance in reporting results was set at *p* < 0.05 with a 95% confidence interval.

## Results

The analysis comprised 3,580 infants and young children aged 6–23 months from three RDHS surveys, i.e. 1,185 in RDHS 2010, 1,152 in RDHS 2014/2015 and 1,243 in RDHS 2019/2020 (Table 1).

**Table 1.** Distribution of children in the study by 2010, 2014/15, and 2019/20 RDHS.

Variable	Total (n)	Mean age (SD)	Urban girls (n, %)	Urban boys (n, %)	Rural girls (n, %)	Rural boys (n, %)
Children 6–23 month	3580	14.37 (5.15)	271 (7.6)	269 (7.5)	1506 (42.1)	1534 (42.8)
2010 RDHS	1185	14.52 (5.11)	67 (5.7)	69 (5.8)	526 (44.4)	523 (44.1)
2014/15 RDHS	1152	14.26 (5.16)	101 (8.8)	95 (8.2)	482 (41.8)	474 (41.1)
2019/20 RDHS	1243	14.32 (5.17)	103 (8.3)	105 (8.4)	498 (40.1)	537 (43.2)

There was a consistent distribution of children across urban and rural settings over the years. Specifically, the proportion of urban girls remained relatively stable, ranging from 5.7% in 2010 to 8.8% in 2014/15 and 8.3% in 2019/20. Similarly, the proportion of urban boys ranged from 5.8% in 2010 to 8.2% in 2014/15 and 8.4% in 2019/20. In contrast, the distribution of rural children was well-represented, with rural girls comprising 44.4% in 2010, dropping to 41.8% in 2014/15, and further to 40.1% in 2019/20. Rural boys comprised 44.1% in 2010, 41.1% in 2014/15, and 43.2% in 2019/20. The mean ages of children across the surveys were also consistent, with slight variations: 14.52 months (SD = 5.11) in 2010, 14.26 months (SD = 5.16) in 2014/15, and 14.32 months (SD = 5.17) in 2019/20. Therefore, the age distribution of children in the study population was stable across the years.

The characteristics of children aged 6–23 months, their mothers and households in Rwanda per year of the RDHS survey are presented in Table 2.

**Table 2.** Characteristics of children and their caregivers in Rwanda by year of survey (weighted)*.

Variables	RDHS 2010 (*n* = 1185)		RDHS 2014/15 (*n* = 1152)		RDHS 2019/20 (*n* = 1243)	
	n	%	n	%	n	%
Child age (in months)						
6–11	399	33.7	409	35.5	439	35.3
12–23	786	66.3	743	64.5	804	64.7
Child’s sex						
Female	593	50.0	583	50.6	601	48.4
Male	592	50.0	569	49.4	642	51.6
Birth size^a^						
Very small	23	2.0	26	2.2	26	2.1
Small	156	13.2	150	13.0	178	14.4
Average/Larger	1002	84.9	974	84.74	1030	83.5
Breastfeeding initiation after birth^b^						
Within first hour	858	72.9	929	80.8	1069	86.3
1–24 hours	269	22.9	184	16.0	144	11.6
1–30 days	49	4.2	38	3.3	26	2.1
Currently breastfeeding						
No	95	8.0	77	6.7	97	7.8
Yes	1090	92.0	1075	93.3	1146	92.2
Minimum dietary diversity (MDD)						
Inadequate	900	75.9	838	72.8	822	66.1
Adequate	285	24.1	314	27.2	421	33.9
Minimum meal frequency (MMF)						
Inadequate	570	48.1	600	52.1	695	55.9
Adequate	615	51.9	552	48.0	548	44.1
Minimum acceptable diet (MAD)						
Inadequate	979	82.6	941	81.7	986	79.3
Adequate	206	17.4	211	18.3	257	20.7
Child given vitamin A rich fruits^c^						
No	911	77.2	892	77.8	706	56.8
Yes	268	22.8	254	22.2	537	43.2
Child received drugs for intestinal parasites in last 6 months^d^						
No	373	31.5	428	37.2	489	39.3
Yes	810	68.5	723	62.8	754	60.7
Had diarrhoea in past 2 weeks						
No	897	75.7	900	78.1	933	75.1
Yes	288	24.3	252	21.9	310	24.9
Had fever in past 2 weeks^e^						
No	902	76.1	862	74.9	904	72.8
Yes	283	23.9	289	25.1	339	27.3
Had cough in past 2 weeks^f^						
No	821	69.3	733	63.7	786	63.3
Yes	364	30.8	418	36.3	456	36.7
Type of birth						
Singleton	1161	98.0	1134	98.5	1191	95.8
Multiple birth	24	2.0	18	1.5	52	4.2
Mother’s age (in years)						
<20	26	2.2	44	3.8	30	2.4
20–34	894	75.5	861	74.8	842	67.8
35–49	265	22.4	247	21.5	371	29.9
Mother’s education status						
No education	208	17.5	142	12.3	121	9.7
Primary	870	73.4	853	74.1	788	63.4
Secondary/Higher	107	9.1	157	13.7	334	26.9
Maternal body mass index (BMI)(kg/m^2^)						
Underweight (BMI <18.5)	62	5.2	55	4.7	51	4.1
Normal (BMI ≥18.5 & ≤24.9)	927	78.2	833	72.3	860	69.2
Overweight (BMI >24.9 & ≤29.9)	172	14.5	211	18.3	239	19.3
Obese (BMI >29.9)	24	2.0	53	4.7	93	7.5
Maternal anaemia status^g^						
Anaemic	201	17.0	206	18.0	141	11.3
Not anaemic	983	83.0	944	82.1	1101	88.7
Iron supplementation during pregnancy						
No	233	19.7	234	20.3	220	17.7
Yes	920	77.7	888	77.1	974	78.4
Missing	32	2.7	30	2.6	49	4.0
Received drugs for intestinal parasites during pregnancy						
No	679	57.3	536	46.5	669	53.9
Yes	466	39.3	584	50.7	523	42.1
Missing	40	3.4	32	2.8	51	4.1
Place of Residence						
Urban	136	11.4	196	17.0	208	16.7
Rural	1049	88.6	956	83.0	1035	83.3
Province						
Kigali City	105	8.9	129	11.2	174	14.0
South	273	23.0	263	22.9	275	22.1
West	330	27.9	269	23.3	286	23.0
North	182	15.3	167	14.5	185	14.9
East	295	24.9	324	28.1	323	26.0
Cluster altitude						
<1500 m	313	26.4	373	32.4	394	31.7
1500-2000 m	628	53.0	578	50.2	651	52.4
>2000 m	244	20.6	201	17.5	198	15.9
Household wealth index						
Poorest	271	22.9	287	24.9	267	21.5
Poorer	276	23.3	248	21.6	267	21.5
Middle	230	19.4	222	19.3	250	20.1
Richer	211	17.8	201	17.5	248	20.0
Richest	197	16.7	194	16.8	211	17.0
Source of drinking water in the household^h^						
Improved	823	70.24	836	73.38	568	66.8
Not improved	349	29.76	303	26.62	282	33.2
Type of toilet facility in the household^i^						
Improved	862	73.7	824	72.3	929	75.9
Not improved	308	26.3	316	27.7	295	24.1

*All frequencies and percentages were weighted.

RDHS: Rwanda Demographic and Health Survey, MDD: Minimum dietary diversity, MMF: Minimum meal frequency, MAD: Minimum acceptable diet.

^a^*n* = 1181 for RDHS 2010, *n* = 1150 for RDHS 2014/15, *n* = 1234 for RDHS 2019/20.

^b^*n* = 1177 for RDHS 2010, *n* = 1150 for RDHS 2014/15, *n* = 1239 for RDHS 2019/20.

^c^*n* = 1179 for RDHS 2010, *n* = 1146 for RDHS 2014/15, *n* = 1243 for RDHS 2019/20.

^d^*n* = 1183 for RDHS 2010, *n* = 1151 for RDHS 2014/15, *n* = 1243 for RDHS 2019/20.

^e^*n* = 1185 for RDHS 2010, *n* = 1151 for RDHS 2014/15, *n* = 1243 for RDHS 2019/20.

^f^*n* = 1185 for RDHS 2010, *n* = 1150 for RDHS 2014/15, *n* = 1243 for RDHS 2019/20.

^g^*n* = 1184 for RDHS 2010, *n* = 1150 for RDHS 2014/15, *n* = 1242 for RDHS 2019/20.

^h^*n* = 1172 for RDHS 2010, *n* = 1139 for RDHS 2014/15, *n* = 850 for RDHS 2019/20.

^i^*n* = 1172 for RDHS 2010, *n* = 1139 for RDHS 2014/15, *n* = 850 for RDHS 2019/20.

Most of the children were aged between 12 and 23 months (66.3% in 2010, 64.5% in 2014/15, and 64.7% in 2019/20), with similar proportions of males and females. Most children had average/larger birth sizes (84.9% in 2010, 84.7% in 2014/15, and 83.5% in 2019/20). Over the period, the proportion of children who received adequate MDD increased from 24.1% in 2010 to 33.9% in 2019/20, while the proportion of children who had received adequate MMF declined from 51.9% in 2010 to 44.1% in 2019/20. The proportion of children who were provided adequate MAD increased from 17.4% in 2010 to 20.7% in 2019/20 (Table 2).

The majority of all the children’s mothers were aged between 20 and 34 years (75.5% in 2010, 74.8% in 2014/15, and 67.8% in 2019/20). During the period, mothers with no education declined from 17.5% in 2010 to 9.7% in 2019/20, while the proportion of mothers with secondary/higher education increased from 9.1% to 26.9%. The proportion of mothers who were underweight decreased from 5.2% in 2010 to 4.1% in 2019/20, while mothers with overweight and obesity has increased from 16.5% to 26.8%. The magnitude of maternal anaemia changed over the three surveys, with 17%, 18%, and 11.3% of all mothers anaemic in 2010, 2014/15, and 2019/20, respectively. The majority of children were from families residing in rural areas, and a large proportion of households had improved drinking water sources and toilet facilities (Table 2).

**Figure 1.**
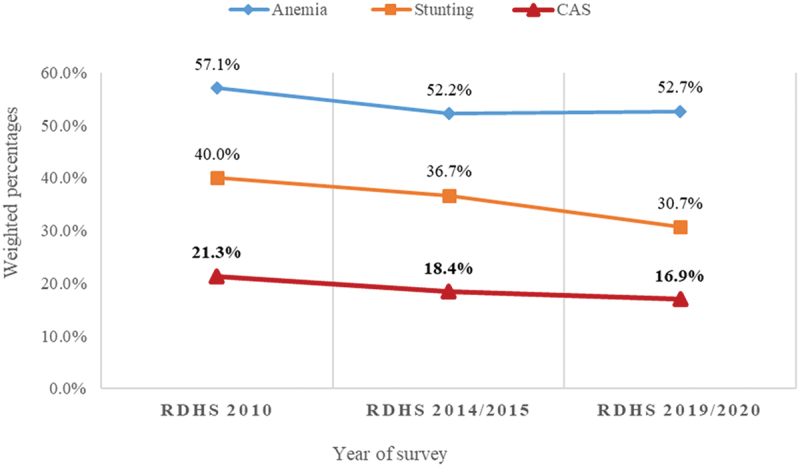
Trend in prevalence of CAS among infants and young children aged 6 to 23 months in Rwanda from 2010 to 2020.

Figure 1 presents CAS prevalence trends (2010 to 2020) in infants and young children.

The prevalence of CAS among infants and young children in Rwanda fell by 4.4% between 2010 and 2020. The prevalence of CAS among these children significantly declined from 21.3% in the 2010 RDHS to 18.4% in the 2014/2015 RDHS and 16.9% 2019/2020 RDHS (*p* = 0.005, 95 CI: 0.0049–0.0058) (Figure 1).

Table 3 summarizes the critical values and significance of the difference in the three prevalence rates of CAS from the RDHS of 2010, 2014/2015, and 2019/2020.

**Table 3.** Critical values and significance of the difference in the three CAS prevalence rates in the 2010, 2014/2015, and 2019/2020 RDHS.

RDHS	Prevalence of CAS (pi)	Absolute Differences (pi-pj)	Value	Critical range	Significant
2010	p1 = 0.2132	[p1-p2]	0.029	0.040	No
2014/15	p2 = 0.1840	[p2-p3]	0.015	0.039	No
2019/20	p3 = 0.1691	[p1-p3]	0.044	0.040	Yes

Analysis of RDHS 2010, 2014/2015, and 2019/2020.

No statistical significance was found in the differences between CAS prevalence in the 2010 RDHS and 2014/2015 RDHS and between the 2014/2015 RDHS and 2019/2020 RDHS. The only difference in the CAS prevalence in the 2010 RDHS compared to the 2019/2020 RDHS was statistically significant (Table 3).

The results from the bivariate analysis of the prevalence of CAS among infants and young children aged 6–23 months by characteristics for each RDHS are presented in Table 4.

**Table 4.** Prevalence of CAS among infants and young children aged 6–23 months according to characteristics, RDHS 2010–2020*.

Variables	RDHS 2010			RDHS 2014/15			RDHS 2019/20		
	CAS (%)	(95%CI)	*p*-value	CAS (%)	(95%CI)	*p*-value	CAS (%)	(95%CI)	*p*-value
Child’s age (in months)			0.005			0.015			0.722
6–11	16.4	[13.1, 20.4]		14.3	[11.1, 18.3]		16.3	[12.7, 20.8]	
12–23	23.8	[20.8, 27.2]		20.6	[17.7, 24.0]		17.2	[14.6, 20.3]	
Child’s sex			<0.001			0.012			0.001
Female	17.2	[14.2, 20.6]		15.4	[12.5, 18.7]		12.9	[10.2, 16.2]	
Male	25.4	[22.2, 29.0]		21.5	[18.1, 25.3]		20.6	[17.4, 24.3]	
Birth size			0.063			0.004			0.002
Very small	36.4	[19.0, 58.1]		30.3	[15.2, 51.5]		41.2	[22.3,63.1]	
Small	26.4	[19.4, 34.8]		27.4	[20.4, 35.7]		21.4	[15.5, 28.7]	
Average/Larger	20.2	[17.8, 22.8]		16.7	[14.4, 19.4]		15.6	[13.4, 18.2]	
Breastfeeding initiation after birth			0.214			0.586			<0.001
Within first hour	20.1	[17.5, 23.1]		17.8	[15.3, 20.7]		16.7	[14.3, 19.3]	
1–24 hours	25.4	[20.3, 31.3]		20.9	[15.4, 27.7]		12.3	[7.8, 19.0]	
1–30 days	23.1	[12.9, 38.1]		21.4	[11.0, 37.5]		50.4	[28.3, 72.4]	
Currently breastfeeding			0.742			0.436			0.948
No	19.9	[12.4, 30.4]		21.9	[13.7, 33.2]		17.2	[10.2, 27.6]	
Yes	21.4	[19.1, 24.0]		18.1	[15.8, 20.7]		16.9	[14.6, 19.4]	
MDD			0.856			0.058			0.039
Inadequate	21.4	[18.9, 24.2]		19.9	[17.1, 23.1]		18.8	[16.1, 21.8]	
Adequate	20.9	[16.3, 26.5]		14.4	[10.7, 19.1]		23.3	[9.7, 17.9]	
MMF			0.289			0.609			
Inadequate	22.6	[19.2, 26.4]		19.0	[15.9, 22.6]		17.9	[15.0, 21.1]	
Adequate	20.1	[17.2, 23.4]		17.7	[14.6, 21.4]		15.7	[12.7, 19.2]	
MAD			0.893						0.019
Inadequate	21.4	[18.9, 24.1]		20.0	[17.3, 22.9]		18.2	[15.7, 21.0]	
Adequate	20.9	[15.5, 27.7]		11.5	[7.8, 16.6]		12.1	[8.6, 16.8]	
Child given vitamin A rich fruits			0.170			0.070			0.069
No	22.2	[19.5, 25.1]		19.5	[16.9, 22.3]		18.8	[15.9, 22.1]	
Yes	18.1	[13.9, 23.4]		13.7	[9.5, 19.4]		14.5	[11.3, 18.3]	
Child received drugs for intestinal parasites in last 6 months			0.448			0.985			0.823
No	19.8	[15.9, 24.5]		18.45	[14.9, 22.6]		17.25	[13.7, 21.5]	
Yes	22.0	[19.0, 25.4]		18.4	[15.6, 21.5]		16.7	[14.0, 19.8]	
Had diarrhea in past 2 weeks			0.323			0.200			0.200
No	20.7	[18.2, 23.5]		17.6	[15.1, 20.3]		16.1	[13.6, 19.0]	
Yes	23.2	[18.9, 28.3]		21.4	[16.4, 27.4]		19.4	[15.3, 24.3]	
Had fever in past 2 weeks			0.096			0.511			0.711
No	20.1	[17.4, 23.0]		18.9	[16.3, 21.8]		16.6	[14.1, 19.6]	
Yes	25.3	[20.2, 31.1]		17.0	[12.7, 22.4]		17.6	[13.5, 22.7]	
Had cough in past 2 weeks						0.122			0.003
No	21.2	[18.4, 24.2]		19.8	[17.0, 23.0]		14.1	[11.6, 17.1]	
Yes	21.7	[17.9, 26.0]		16.0	[12.6, 20.0]		21.7	[17.7, 26.5]	
Type of birth			0.170			0.283			0.003
Singleton	21.0	[18.7, 23.6]		18.2	[16.0, 20.7]		16.0	[13.9, 18.3]	
Multiple birth	35.0	[16.3, 59.9]		31.7	[10.6,64.6]		37.5	[21.4, 57.0]	
Mother’s age (in years)			0.001			0.918			0.131
<20	47.5	[30.7, 65.0]		19.1	[9.9, 33.8]		6.8	[2.0, 20.5]	
20–34	19.6	[16.9, 22.5]		18.1	[15.5, 21.0]		16.2	[13.8, 19.0]	
35–49	24.7	[19.7, 30.4]		19.3	[14.4, 25.3]		19.3	[15.1, 24.3]	
Mother’s education status			0.001			0.027			0.001
No formal education	26.2	[20.6, 32.7]		23.6	[17.2, 31.55]		27.4	[19.7, 36.7]	
Primary	21.8	[19.0, 24.9]		18.8	[16.2, 21.8]		17.5	[14.6, 20.9]	
Secondary/Higher	8.2	[4.4, 14.5]		11.4	[7.3, 17.6]		11.7	[8.6, 15.7]	
Maternal body mass index (BMI)(kg/m^2^)			0.059			0.175			0.008
Underweight (BMI <18.5)	12.6	[6.4,23.3]		18.1	[9.9, 30.8]		26.5	[16.1, 40.4]	
Normal (BMI ≥18.5 & ≤24.9)	23.0	[20.3, 26.0]		20.0	[17.2, 23.0]		18.7	[15.9, 21.9]	
Overweight (BMI >24.9 & ≤29.9)	16.1	[10.9, 23.0]		14.2	[9.3, 21.1]		12.3	[8.4, 17.6]	
Obese (BMI >29.9)	15.4	[6.0, 34.0]		10.85	[4.9, 22.2]		7.1	[2.8, 16.9]	
Maternal anaemia status			0.083			<0.001			0.102
Anaemic	26.3	[20.1, 33.7]		27.8	[21.1, 35.7]		21.6	[15.8, 28.9]	
Not anaemic	20.3	[17.9, 23.0]		16.3	[14.0, 19.0]		16.3	[14.0, 19.0]	
Iron supplementation during pregnancy			0.028			0.332			0.600
No	26.7	[20.9, 33.3]		16.1	[11.7, 21.7]		18.05	[13.2, 24.2]	
Yes	19.6	[17.2, 22.3]		19.0	[16.5, 21.8]		16.5	[14.1, 19.2]	
Received drugs for intestinal parasites during pregnancy			0.141			0.103			0.229
No	19.4	[16.6, 22.6]		20.55	[17.3, 24.3]		18.0	[15.1, 21.4]	
Yes	23.1	[19.4, 27.3]		16.5	[13.5, 20.1]		15.2	[12.2, 18.8]	
Place of Residence			0.112			0.103			0.001
Urban	15.6	[10.0, 23.3]		14.45	[10.5, 19.6]		8.7	[5.3, 13.8]	
Rural	22.1	[19.5, 24.8]		19.2	[16.7, 22.0]		18.6	[16.0, 21.4]	
Province			0.013			0.098			0.023
Kigali City	13.5	[8.3, 21.4]		10.2	[6.2, 16.3]		13.0	[8.1, 20.3]	
South	18.4	[14.1, 23.7]		20.1	[15.7, 25.4]		14.8	[10.3, 20.7]	
West	19.5	[15.5, 24.1]		21.6	[17.4, 26.5]		19.5	[15.1, 24.9]	
North	23.0	[16.9, 30.3]		18.3	[12.9, 25.3]		24.9	[18.9, 31.9]	
East	27.9	[22.8, 33.5]		17.65	[13.2, 23.2]		14.0	[10.4, 18.5]	
Cluster altitude						0.007			0.003
<1500 m	21.3	[17.1, 26.4]		15.6	[11.7, 20.5]		13.3	[10.0, 17.4]	
1500-2000 m	19.5	[16.2, 23.2]		17.35	[14.5, 20.6]		16.6	[13.5, 20.4]	
>2000 m	26.0	[21.3, 31.4]		26.6	[21.0, 33.0]		25.0	[20.3, 30.5]	
Household wealth index			0.016			0.011			<0.001
Poorest	25.5	[20.6, 31.1]		23.4	[18.7, 28.8]		26.0	[21.3, 31.4]	
Poorer	24.4	[20.0, 29.4]		22.6	[17.4, 28.9]		21.4	[16.4, 27.4]	
Middle	21.1	[15.9, 27.6]		15.1	[10.8, 20.8]		14.2	[9.4, 21.1]	
Richer	19.5	[14.8, 25.3]		15.9	[10.7, 22.9]		14.1	[9.5, 20.3]	
Richest	13.4	[9.4, 18.8]		12.0	[8.2, 17.12]		6.1	[3.4, 10.8]	
Source of drinking water in the household			0.081			0.056			0.084
Improved	19.9	[17.2, 23.0]		17.0	[14.5, 19.9]		14.6	[11.5, 18.3]	
Not improved	24.8	[20.2, 29.9]		22.7	[17.7, 28.6]		19.4	[15.1, 24.6]	
Type of toilet facility in the household			0.003			0.153			0.222
Improved	19.1	[16.6, 22.0]		17.4	[14.8, 20.3]		16.25	[13.6, 19.3]	
Not improved	27.3	[22.5, 32.7]		21.4	[16.9, 26.7]		19.5	[15.4, 24.4]	

*All percentages were weighted.

CAS: Concurrent anaemia and stunting; RDHS: Rwanda Demographic and Health Survey; MDD: Minimum dietary diversity; MMF: Minimum meal frequency; MAD: Minimum acceptable diet.

The prevalence of CAS was significantly higher in children over 12 months of than in infants aged 6–11 months in 2010 (23.8% vs. 16.4%, *p* = 0.005) and in 2014/15 (20.6% vs. 14.3%, *p* = 0.015), while the prevalence was similar in 2019/20 in these two age groups. The prevalence of CAS was significantly higher in boys across all years, 25.4% in 2010 (*p* < 0.001), 21.5% in 2014/15 (*p* = 0.012), and 20.6% in 2019/20 (*p* = 0.001). The CAS prevalence was higher in children with very small or small birth sizes compared with the average and above (30.3% and 27.4% vs 16.7% in 2014/15, and 41.2% and 21.4% vs 15.6% in 2019/20). The prevalence of CAS was also significantly higher in infants and young children who were initiated to breastfeeding between 1 and 30 days after birth compared to their counterparts who had breastfeeding initiation within the first hour or one to 24 h after birth in 2019/20 (50.4% vs. 16.7% and 12.3%, respectively, *p* < 0.001). In contrast, there were no differences in the prevalence of CAS in children who stated breastfeeding up to 1 day after birth in 2010 and 2014/15. Additionally, CAS was significantly more prevalent among infants and young children who received inadequate MAD compared to those who received adequate MAD (20% vs. 11.5% in 2014/15 and 18.2% vs. 12.1% in 2019/20, respectively) (Table 4).

Children of mothers under 20 years had a high prevalence rate of CAS (47.5%) in 2010, compared to 19.6% and 24.7% in children of mothers aged 20–34 years and 35–49 years, respectively. However, there were no significant differences in the prevalence rate of CAS in children between mothers’ age groups in 2014/2015 and 2019/2020. The mother’s education status was associated with CAS across the three surveys. Children of mothers with no education had higher prevalence of CAS than those from mothers with primary and secondary/higher education. The prevalence of CAS was higher in children from mothers with anaemia compared to children of mothers without anaemia. However, this was only significant in 2014/15 (27.8% versus 16.3%, respectively, *p* < 0.001) (Table 4).

Across the three surveys, the prevalence of CAS was significantly more prevalent among children from the poorest and poorer households. In addition, children from households with an unimproved source of drinking water and those from households with unimproved toilet facilities had higher CAS prevalence than their counterparts from households with improved drinking water and toilet facilities. However, the differences were only significant in 2010 for the type of toilet facility (Table 4).

Table 5 summarizes findings from binary logistic regression analysis that was conducted to investigate factors associated with CAS in infants and young children in Rwanda for the 2010, 2014/2015, and 2019/2020 RDHS.

**Table 5.** Factors associated with CAS in infants and young children aged 6–23 months children in Rwanda.

Variables	RDHS 2010		RDHS 2014/15		RDHS 2019/20	
	Full model	Final model	Full model	Final model	Full model	Final model
	AOR (95%CI)	AOR (95%CI)	AOR (95%CI)	AOR (95%CI)	AOR (95%CI)	AOR (95%CI)
Child age (in months)						
6–11	ref	ref	ref	ref		
12–23	1.59 (1.13–2.25)	1.58 (1.11–2.23)*	1.62 (1.13–2.34)	1.62 (1.13–2.31)**		
Child’s sex						
Female	ref	ref	ref	ref	ref	ref
Male	1.67 (1.24–2.25)	1.67 (1.23–2.25)**	1.44 (1.04–1.99)	1.48 (1.07–2.04)*	1.82 (1.28–2.58)	1.79 (1.27–2.53)**
Birth size						
Very small			1.92 (0.79–4.68)	2.02 (0.83–4.88)	3.44 (1.33–8.86)	3.10 (1.23–7.78)*
Small			1.83 (1.16–2.88)	1.89 (1.22–2.94)*	1.43 (0.91–2.24)	1.46 (0.93–2.29)
Average/Larger			ref	ref	ref	ref
Breastfeeding initiation after birth						
Within first hour					ref	ref
1–24 hours					0.66 (0.35–1.25)	0.68 (0.36–1.26)
1–30 days					5.45 (2.03–14.63)	6.26 (2.1–16.30)***
MDD						
Inadequate					1.16 (0.63–1.74)	
Adequate					ref	
MAD						
Inadequate			1.63 (1.00–2.67)	1.80 (1.11–2.91)*	0.89 (0.45–1.74)	
Adequate			ref	ref	ref	
Had cough in past 2 weeks						
No					ref	ref
Yes					1.55 (1.08–2.23)	1.60 (1.12–2.27)*
Type of birth						
Singleton					ref	ref
Multiple birth					2.92 (1.12–7.59)	3.08 (1.27–7.46)*
Mother’s age (in years)						
<20	3.91 (1.50–10.14)	3.99 (1.52–1048)**				
20–34	ref	ref				
35–49	1.22 (0.83–1.79)	1.21 (0.83–1.77)				
Mother’s education status						
No education	3.58 (1.51–8.49)	4.07 (1.80–9.21)**	1.49 (0.73–3.01)		1.47 (0.79–2.71)	
Primary	2.75 (1.22–6.22)	3.03 (1.40–6.56)**	1.32 (0.75–2.32)		1.09 (0.67–1.77)	
Secondary/Higher	ref	ref	ref		ref	
Maternal BMI						
Underweight					1.50 (0.73–3.09)	
Normal BMI					ref	
Overweight					0.86 (0.53–1.37)	
Obese					0.63 (0.21–1.85)	
Maternal anaemia status						
Anaemic			1.89 (1.23–2.93)	1.95 (1.26–3.02)**		
Not anaemic			ref	ref		
Iron supplementation during pregnancy						
No	1.47 (1.0–2.17)	1.50 (1.02–2.21)*				
Yes	ref	ref				
Place of Residence						
Urban					ref	
Rural					1.37 (0.72–2.62)	
Province						
Kigali City	ref	ref			ref	
South	0.88 (0.41–1.88)	1.07 (0.53–2.16)			0.63 (0.31–1.24)	
West	0.95 (0.44–2.03)	1.11 (0.54–2.25)			0.84 (0.43–1.62)	
North	1.34 (0.60–2.97)	1.54 (0.73–3.27)			1.14 (0.57–2.27)	
East	1.76 (0.85–3.61)	1.97 (1.00–3.87)*			0.76 (0.38–1.51)	
Cluster altitude						
<1500 m			ref	ref	ref	ref
1500-2000 m			1.14 (0.75–1.75)	1.78 (0.78–1.77)	1.0 (0.63–1.59)	1.12 (0.73–1.71)
>2000 m			1.77 (1.08–2.88)	1.94 (1.22–3.09)**	1.53 (0.87–2.69)	1.78 (1.12–2.84)*
Household wealth index						
Poorest	1.54 (0.83–2.86)		1.35 (0.75–2.43)		3.45 (1.35–8.85)	4.80 (2.35–9.82)***
Poorer	1.40 (0.77–2.53)		1.38 (0.75–2.52)		2.55 (0.97–6.65)	3.51 (1.65–7.44)**
Middle	1.23 (0.68–2.23)		0.94 (0.52–1.69)		1.94 (0.75–5.07)	2.53 (1.15–5.55)*
Richer	1.11 (0.612–2.0)		1.20 (0.60–2.40)		2.19 (0.88–5.47)	2.70 (1.22–5.98*
Richest	ref		ref		ref	ref
Type of toilet facility in the household						
Improved	ref	ref				
Not improved	1.46 (1.05–2.03)	1.63 (1.20–2.21)**				

CAS: Concurrent anaemia and stunting; RDHS: Rwanda Demographic and Health Survey; AOR: Adjusted odd ratios; ref: reference; MDD: Minimum dietary diversity; MAD: Minimum acceptable diet; BMI: Body mass index.

*p-value: <0.005, **p-value: <0.01, *p*-value: <0.0001.

Overall, the associations between CAS in infants and young children aged 6–23 months and different participants’ characteristics varied. Across the three surveys, male children had 1.23 to 1.79 times higher odds of CAS than female children. Children aged 12–23 months had higher odds of CAS in 2010 and 2014/15 (AOR: 1.58, 95% CI: 1.11–2.23 and 1.62, 95% CI: 1.13–2.31, respectively). Small birth size and very small birth size were associated with higher odds of CAS in 2014/15 and 2019/20 (AOR: 1.89, 95% CI: 1.22–2.94 and AOR: 3.1, 95% CI: 1.23–7.78), respectively. In 2014/15, children who had received inadequate MAD had a greater likelihood of CAS (AOR: 1.80, 95% CI: 1.11–2.91). In 2019/20, the odds of CAS were significantly associated with the following factors: breastfeeding initiation within 1 day after birth (AOR: 6.26, 95% CI: 2.1–16.30), a history of cough in the 2 weeks prior to the survey (AOR: 1.6, 95% CI: 1.12–2.27), and the child being one of multiple births (AOR: 3.08, 95% CI: 1.27–7.46) (Table 5).

In 2010, children whose mothers were below 20 years had higher odds of CAS (AOR: 3.91, 95% CI: 1.50–10.14) compared to those whose mothers were older than 20 years. Compared to secondary/higher education level, children whose mothers did not attend any school (AOR: 3.58, 95% CI: 1.51–8.49) or only achieved primary education level (AOR: 2.75, 95% CI: 1.22–6.22) had significantly higher likelihoods of CAS. The odds of CAS were significantly higher in children from mothers who did not take iron supplementation during pregnancy (AOR: 1.50, 95% CI: 1.02–2.21). Maternal anaemia was significantly associated with higher odds of CAS in 2014/15 (AOR: 1.95, 95% CI: 1.26–3.02) (Table 5).

Children from the eastern province significantly had 1.97 times higher odds of CAS compared with children from Kigali city in 2010 (95% CI: 1.00–3.87). Compared to children from clusters with altitudes less than 1500 m, the odds of CAS were significantly 1.94 times (95% CI: 1.22–3.09) and 1.78 times (95% CI: 1.12–2.84) higher in children from cluster altitudes higher than 2000 m in 2014/15 and 2019/20, respectively. The household wealth index was significantly associated with CAS in 2019/20: the lower the wealth index, the higher the odds of CAS increasing. Compared to children from the wealthiest households, children from households with the poorest and poorer wealth indexes had significantly higher odds of CAS (AOR: 4.80, 95% CI: 2.35–9.82 and AOR: 3.51, 95% CI: 1.65–7.44, respectively). Additionally, in 2010, the likelihood of CAS was significantly higher for children from households with unimproved toilet facilities compared to those from households with improved toilet facilities (AOR: 1.63, 95% CI: 1.20–2.21) (Table 5).

## Discussion

This study sought to explore the magnitude of, and factors associated with, CAS in Rwanda. The findings reveal significant reductions in the prevalence of CAS among children aged 6–23 months during the past decade. We found that CAS is linked to different factors which varied across the three surveys. In particular, we found that child’s age above 12 months, being male, having a very small or small birth size, breastfeeding initiation within 1 day after birth, receiving inadequate MAD, history of cough in 2 weeks prior to the survey interview, maternal age below 20 years, no iron supplementation during pregnancy, maternal anaemia, resident of the eastern province, living in clusters with altitudes above 2000 m, being from households with low wealth, and unimproved types of toilets, were all factors associated with CAS in infants and young children in Rwanda.

The prevalence of CAS decreased significantly from 21.3% in 2010 to 18.4% in 2014/2015 and further to 16.9% in 2019/2020. This trend indicates substantial improvements in the nutritional and health status of young children in Rwanda. These results are consistent with trends observed in similar studies in other countries. For instance, Black et al. [33] reported a global decline in stunting and anaemia due to enhanced nutritional interventions and public health strategies, while Stevens et al. [34] noted significant reductions in anaemia prevalence among children under five worldwide and attributed this to improved dietary practices and iron supplementation programs. In contrast, some regions have reported less pronounced declines. Akombi et al. [35] highlighted that, despite improvements, the rates of stunting and anaemia remain high in Sub-Saharan Africa due to persistent socio-economic challenges and limited healthcare access. This underscores the importance of contextual factors such as economic stability, healthcare infrastructure, and public health policies in influencing CAS prevalence.

Despite the overall decline in CAS rates, the prevalence of anaemia alone remains high and exceeds global rates (>40%) [3,6]. Our study found no statistically significant differences in CAS prevalence between the 2010 and 2014/2015 RDHS, or between the 2014/2015 and 2019/2020 RDHS. However, the difference between the 2010 and 2019/2020 RDHS was significant, indicating a notable long-term decline over the decade. This pattern aligns with the findings from other studies [33,34] which observed gradual improvements in child health indicators over extended periods rather than dramatic shifts in shorter intervals. The prevalence rates found in our present study are lower compared to rates reported from other countries, such as 25% in 22 Sub-Saharan African countries [19], 21.5% in 43 low- and middle-income countries (18), and higher compared to Ghana’s 10.6% [36]. Our findings highlight that multiple forms of undernutrition remain a significant public health concern in Rwanda, underscoring the need for targeted strategies to address both anaemia and stunting concurrently.

This study identified different factors associated with CAS and found that they varied across the three surveys. In line with evidence from Ethiopia [20], children aged over 12 months were more likely to be anaemic and stunted at the same time together compared to those aged 6–11 months. This may be because undernutrition in developing countries is more prevalent in the early years of childhood and peaking high in the second year of life [37,38]. Male children across the three surveys had higher odds of developing CAS compared to females. This is consistent with the results from studies carried out in Ethiopia [20] and in 43 developing countries [18]. Compared to children born with average/larger birth sizes, in the present study children born with very small and small birth sizes were more likely to have CAS. Small birth size indicates intrauterine malnutrition, which is linked with inadequate nutritional intake and maternal malnutrition during pregnancy [20]. This finding suggests the need to improve integrated nutritional interventions during pregnancy and establish specific interventions to support infants with small birth sizes in achieving growth catch-up.

Children with poor feeding practices, imbalanced diets, and inadequate intake of micronutrients are highly affected by multiple forms of undernutrition [19,39]. In this study, infants and children with delayed breastfeeding initiation (one to 30 days after birth) and inadequate MAD had higher likelihoods of CAS. This result aligns with the findings reported by Christian, Afful‑Dadzie, and Marquis [19]. However, contrary to the findings from the latter study, meal frequency and food diversity were not found to be associated with CAS. Both timely early breastfeeding initiation and appropriate feeding practices are among the direct, immediate determinants of child nutrition [16,39]. Therefore, anaemia, stunting, and their coexistence in children can be significantly prevented by implementing combined interventions, with the promotion of early breastfeeding initiation and enhanced complementary feeding practices with adequate dietary diversity in the first years of life [40]. In the current study, infants and young children who had a history of cough 2 weeks before the survey interview were more likely to develop CAS. This finding consistently aligns with previous studies in India and Peru [10] and Ethiopia [20]. Coughing in children is often a symptom of infections, which are predictors of childhood anaemia [2]. This suggests a need to strengthen programs that focus on prevention, early detection, and managing childhood infections.

In this study, maternal age was associated with CAS in children. Infants and young children whose mothers were younger than 20 years had significantly higher odds of acquiring CAS. Like the results from Ethiopia [30], children whose mothers did not take iron supplements during pregnancy were more prone to having CAS. There is evidence that iron supplementation and an adequate intake of other micronutrients reduce maternal malnutrition and prevent maternal anaemia [17,40]. As expected, infants and children born to anaemic mothers were more prone to developing CAS. Poor nutrition during pregnancy and lactation is linked to childhood stunting [16,39,40] and anaemia [7,41]. Strategies focusing on reducing maternal anaemia need to be strengthened.

Unlike previous studies in which living in rural areas was reported as a factor associated with CAS [18,20], this was not found to be a significant factor in the present study. However, residence was found to be associated with CAS, whereby infants and children from the eastern province of Rwanda had a significantly higher likelihood of CAS. The disparity among provinces requires further research. CAS was more likely to affect children from regions with high altitudes, more than 2000 m. Consistent with evidence from other studies [18–20], infants and young children from poor homes had greater likelihoods of developing CAS. In this study, the odds of CAS increased with lower household wealth, with the poorest families having the highest likelihood of CAS in their children. Furthermore, children from families with unimproved toilets had higher odds of having CAS. This is in consistent with findings from previous studies in Sub-Saharan Africa [19]. The results from the present study suggest a need to strengthen existing multi-sectoral interventions aimed at reducing poverty in households, empowering families to achieve socio-economic resilience, and improving sanitation conditions at home.

For nutrition and public health researchers, our findings highlight the complexity of addressing CAS and the necessity for sustained comprehensive intervention strategies. Short-term changes were insignificant, emphasizing the need for extended follow-up periods when evaluating public health interventions. Policymakers should advocate for continuous funding and support for nutritional and health programs, emphasizing long-term commitment to improving child health outcomes. Our study extends previous research by providing updated, region-specific data on prevalence and factors associated with CAS in infants and young children. While global studies have shown gradual improvements in child health indicators, our research underscores the importance of long-term perspectives to identify significant changes. By highlighting the slow yet steady decline in CAS, we contribute to a broader understanding of how sustained interventions impact child health.

## Strengths and weaknesses of the study

Our study has the following strengths. The study leverages three recent RDHS datasets (2010, 2014/2015, and 2019/2020). With a sample size of 3580 infants and young children across three survey periods, the study benefits from a large and representative population. This enhances the generalizability of the findings to the broader Rwandan child population. By examining CAS, the study addresses a critical and multifaceted public health issue. This dual-focus approach highlights the compounded impact of multiple forms of malnutrition, which is more informative for policy and intervention strategies. The study reports local trends and situates these findings within the global context. This comparative analysis helps identify unique local challenges and common global patterns, providing a comprehensive understanding of CAS. The study’s results affect public health policies and interventions. By identifying factors associated with CAS and discussing the effectiveness of past interventions, the study provides actionable insights to guide future policies and programs in Rwanda.

However, the following limitations are acknowledged. The study’s observational nature restricts the ability to draw causal conclusions about the relationship between the interventions and the observed changes in CAS prevalence. While associations can be identified, causality cannot be established. Despite adjusting for various factors, unmeasured confounding variables may influence CAS prevalence. Socio-economic, environmental, and behavioural factors not captured in the RDHS data could affect the results. The reliance on self-reported data collected in interviews can introduce recall bias and social desirability bias. This may affect the accuracy of reported health and nutritional status information, hence potentially influencing the study’s findings. While the findings are highly relevant to Rwanda, their applicability to other countries or regions with different socio-economic and healthcare contexts may be limited. The unique characteristics of Rwanda’s health and nutrition interventions must be considered when extrapolating the results to other settings.

## Conclusions

The concurrence of anaemia and stunting among infants and young children is evident, with approximately 16.9% currently affected. Although there has been a declining trend, the prevalence of CAS among Rwandan children remains a concern, warranting increased public health attention. CAS is associated with various child, maternal, and household-related factors. Our findings underscore that CAS remains a significant public health issue in Rwanda, highlighting the need for targeted strategies and integrated, multi-sectoral interventions.
